# Progress and Clinical Application of Single-Cell Transcriptional Sequencing Technology in Cancer Research

**DOI:** 10.3389/fonc.2020.593085

**Published:** 2021-02-03

**Authors:** Jian Liu, Tianmin Xu, Yuemei Jin, Bingyu Huang, Yan Zhang

**Affiliations:** ^1^ Department of Gynaecology and Obstetrics, Jilin University Second Hospital, ChangChun, China; ^2^ Department of Breast Surgery, Jilin University Second Hospital, ChangChun, China

**Keywords:** scRNA-seq, precision medicine, tumor heterogeneity, tumor microenvironment, cancer cells

## Abstract

Cancer has been a daunting challenge for human beings because of its clonal heterogeneity and compositional complexity. Tumors are composed of cancer cells and a variety of non-cancer cells, which together with the extracellular matrix form the tumor microenvironment. These cancer-related cells and components and immune mechanisms can affect the development and progression of cancer and are associated with patient diagnosis, treatment and prognosis. As the first choice for the study of complex biological systems, single-cell transcriptional sequencing (scRNA-seq) has been widely used in cancer research. ScRNA-seq has made breakthrough discoveries in tumor heterogeneity, tumor evolution, metastasis and spread, development of chemoresistance, and the relationship between the tumor microenvironment and the immune system. These results will guide clinical cancer treatment and promote personalized and highly accurate cancer treatment. In this paper, we summarize the latest research progress of scRNA-seq and its guiding significance for clinical treatment.

## Introduction

The expression measured by traditional bulk transcriptome sequencing (Bulk RNA-seq) is the average expression of hundreds of single cells in the sample, so it is difficult to get the heterogeneity information of the cell population, and those cells that deviate from the average expression level may have important biological significance. Among the many genomic technologies currently available, scRNA-seq is the most useful and reliable method for detecting the biological mechanisms of low-expressing cell populations in tumors (1). Transcriptome sequencing based on the expression level of individual cells has also greatly expanded the application scope of transcriptome research.

Since the single-cell transcriptomic analysis was first reported (2), many other scRNA-seq methods have been developed, such as MARS-seq (Massively parallel RNA single cell sequencing), Smart-seq(Switching mechanism at 5´end of the RNA template sequencing), Cel-seq(Cell expression by linear amplification and sequencing, MATQ-seq (multiple annealing and dC-tailing-based quantitative single-cell RNA-seq), *etc*. The difference between them is mainly the method to amplify the mRNA transcript to produce a full-length cDNA with a unique molecular identity (UMI) at the 5´or 3´end. In order to promote automation and simplify sample preparation, droplet-based ultra-high-throughput single-cell sequencing technology relying on cellular bar coding has emerged since 2015. It is divided into the inDrop (3) and Drop-seq (4) systems developed by David Weitz et al. at Harvard University and the Chromium platform of 10x Genomics Company (5). Based on the new Chromium system of 10x Genomics, thousands or even tens of thousands of single-cell population analyzes can be achieved, thus overcoming the shortcomings of conventional scRNA-seq methods in terms of throughput or scalability, and providing new ideas for single-cell studies (6).

**Figure 1:**
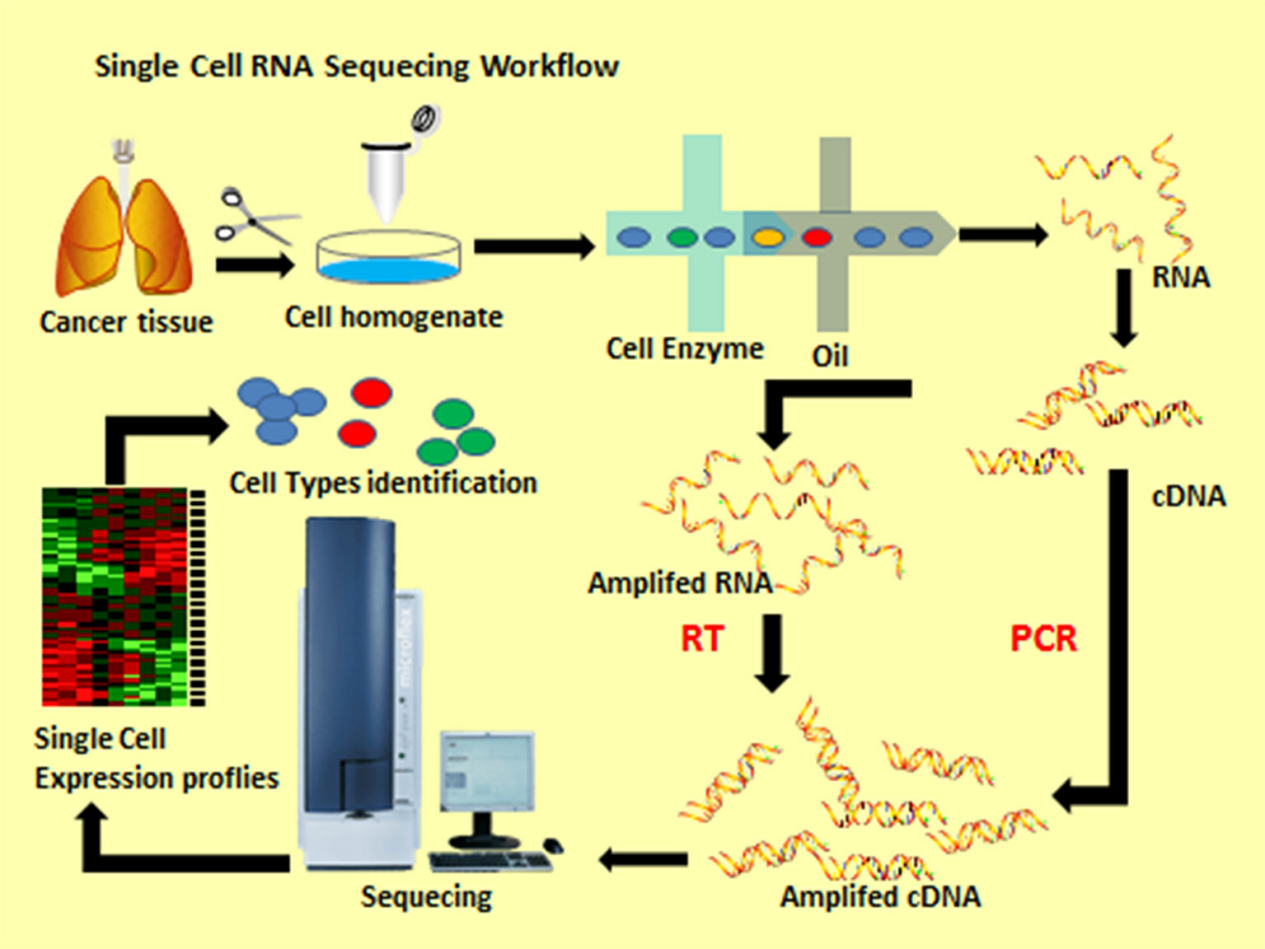
Single cell RNA sequencing workflow.

General scRNA-seq workflow includes many steps (**Figure 1**). All single-cell protocols start with a suspension of cells. For most tissues, this means that beforehand, the extracellular matrix that holds cells together has to be processed to loosen this mesh and to induce the release of cells into suspension. In a lot of cases, dissociation protocols are combined of both approaches by cutting tissues into small pieces, which are then dissociated by an enzymatic treatment. The principle of droplet-based ultra-high-throughput single-cell sequencing technology is built on the microfluidic control system to produce nanoscale droplets of single cells and single microspheres. Each microsphere surface is wrapped with a specific oligonucleotide sequence (cellular barcode) and UMIs to mark the RNA molecules from the same cell (7). Next, cells dissolve in the reaction system and undergo reverse transcription to form full-length cDNA sequences. The minute amounts of cDNA are then amplified by PCR to construct a cDNA library and the Illumina platform are used to sequence it after qualifying (8). In a subsequent step, we use a series of computing tools to process, analyze and visualize these datasets in order to obtain heterogeneity information (9).

ScRNA-seq has been applied in tumor research, such as dividing tumors into different subtypes to explore cell development trajectory (10), constructing a microenvironmental blueprint for tumors (11), discovering new biomarkers and monitoring circulating tumor cells (12), discovering rare cells and mapping them (13), identifying the mechanism of drug resistance and finding new therapeutic targets (14), explaining paracrine signaling networks (15), and making a strategy to develop combined therapy to target multiple related cells in the tumor. ScRNA-seq has been used in a variety of cancer studies (**Table 1**). In this paper, we will present the latest results from cancer studies using 10x Genomics scRNA-seq.

**Table 1:** Summary of the studies in human tumors using scRNAseq.

Cancer type	Tumor cell types	scRNAseq method	Cell number	References	
Follicular B-cell lymphomas	Cancer tissue cells	10×Genomics	34,188	(16)
Breast cancer (TNBC)	CD3+ TILs	10×Genomics and Fluidigm C1	6,311	(17)
Breast cancer (TNBC)	PDX	10×Genomics	3,500	(18)
Breast cancer (TNBC)	M6-Ctrl,M6-Hh cells	10×Genomics	14,950	(19)
Breast cancer	Cancer epithelial cells	10×Genomics and Fluidigm C1	24,646	(20)
Gliomas	Inter (CD11b^+^) and Intra tumor TAMs (*in silico*)	10×Genomics and Fluidigm C1	5,455	(21)
Gastric Premalignant Lesions and Early Gastric Cancer	Cancer epithelial cells	10×Genomics	56,440	(22)
Melanoma	PDX	10×Genomics	8,700	(23)
Melanoma Merkel cell carcinoma (MCC) Leukemia Human kidney tumors Hepatocellular carcinoma (HCC) Hepatocellular carcinoma Hepatocellular carcinoma Pancreatic ductal adenocarcinoma Human glioblastomas	451Lu-Par, 451Lu-BR, A375-BR,451Lu-BR3 cells Cancer tissue cells CMVpp65- or PRAME-specific T cells normal and cancerous kidney cells circulating tumor cells (CTCs) HuH1 and HuH7 cell lines T cells pancreatic cells EGFR wild-type and EGFRvIII mutant cells	10×Genomics 10×Genomics 3’ Chromium 10×Genomics 10×Genomics 10×Genomics 10x Genomics 10×Genomics 10×Genomics 10×Genomics	6,500 11,021 5,000 72,501 7,104 3,847 5,063 57,530 16,128	(24) (25) (26) (27) (28) (29) (30) (31) (32)
Non-small-cell lung cancer (NSCLC)	stromal cells	10×Genomics	84,381	(33)
T-cell acute lymphoblastic leukemia (T-ALL)	T-ALL cells	10×Genomics	2,074	(34)
Alveolar Rhabdomyosarcoma (ARMS) Head and neck squamous cell carcinoma (HNSCC)	circulating tumor cells Cancer tissue cells	10×Genomics 10×Genomics	416 5,902	(35) (36)

## Tumor Cell Heterogeneity Study

Different types of tumors, the same tumor formed by different individuals, and even cancer cells of the same clone are usually heterogeneous, and this heterogeneity can change as the disease progresses (37). ScRNA-seq allows the study of alternative polyadenylation (APA) patterns and gene expression levels of tumor cells in different tumors. In addition, APA and gene expression in specific cell types are consistent, suggesting that cell types can be identified based on changes in the length of 3 ‘untranslated region (3’ UTR) combined with gene expression (38).

### Heterogeneity Study of Subtypes of Tumor Cells

In one study, scRNA-seq was used to identify two catheter subtypes with abnormal and malignant gene expression profiles, respectively, from primary PDAC tumors, and the malignant subtype consisted of several subgroups with different proliferation and migration potentials (31). Interestingly, scRNA-seq indicated that there were three different groups of mammary epithelial cells, and that multiple subclusters were present in each major epithelial cell type (20). At the single-cell level, researchers also found liver CSCs and demonstrated that liver CSCs are heterogeneous in phenotype, function and transcriptome, and that different genes in different CSC are independently associated with the prognosis of hepatocellular carcinoma (HCC). Different oncogenes may drive various CSC subtypes identified by different cell surface markers, which challenge the definition of molecular-targeted therapeutic agents (29). It is controversial whether there are bipotent stem cells in breast. ScRNA-Seq data from human mammary epithelial cells revealed a highly efficient cellular state enriched in independent mammary stem cell expression patterns. Bipotent stem-cell-like cells are associated with the clinical outcome of breast cancer, that is, overexpression of regulatory genes Ybx1 and ENO1 is associated with the risk of breast cancer (39). In addition, assessing the prognostic value of CSC based on single-cell transcriptional data can provide evidence for intratumoral heterogeneity, tumor progression and its clinical significance.

With the application of single-cell technology, researchers have explored and understand transcriptional events in cells of early embryonic development. For example, for the first time, researchers have compared Wilms tumor cells and renal cancer cells with normal renal cells at different developmental stages and ages (fetus, children, adolescents and adults) by scRNA-seq. They found that Wilms tumor cells in pediatric patients share characteristics with specific renal cells in normal development, thus providing evidence for the hypothesis that Wilms tumor cells are abnormal fetal cells. In addition, in adult renal cell carcinoma, researchers have found that renal cancer cells in adult patients originate from a rare healthy adult renal cell PT1 (27). This finding provides a new idea to treat renal cell carcinoma in children by controlling the development of cancer cells rather than killing cancer cells by chemotherapy. These results may lay a foundation for the development of novel treatments for renal cell carcinoma that target PT1 renal cells. Similarly, another study showed that human cerebellar tumor transcriptome is most similar to that in fetal life development. Single-cell transcriptome data highlight cerebellar tumors is a disease of early brain development and provide the most direct evidence for the peak incidence of brain tumors in early childhood (40).

ScRNA-seq was used to construct single-cell maps of cancer cells through analysis data from many samples. For example, scRNA-seq was used to construct a single-cell transcriptome network of cellular and molecular characteristics of gastric epithelial cells with different lesions in a study of EGC (22) and to reveal the biological basis of cell development status of each MB subgroup from 25 medulloblastomas (41). These studies have built some single-cell databases and can be used in future studies. With the help of these new system maps, researchers can lock disease-causing genes in special types of cells, develop new treatments for tumors and assess the chance of tumor metastasis and the impact of treatment on the development of cellular state (42).

### Study on Genetic Heterogeneity and Phenotypic Variation of Tumor Cells

Genotype information deduced from scRNA-seq data can help reveal the heterogeneity of genes and their transcriptions as well as their interaction in tumor progression. The researchers used an integrated method for scRNA-seq to analyze the inheritance, expression, and function of 401 specimens from TCGA and obtained a cellular map of the cellular status and genetic diversity of glioblastoma (13). Studies on gene expression heterogeneity with scRNA-seq in MCF7, a common breast cancer cell line, showed that persistent instability was quickly transformed into cell line heterogeneity. In addition, genetic heterogeneity produces unique gene expression patterns, resulting in differential sensitivity of tumor cells to drugs (43).

At the same time, some studies use scRNA-seq to investigate the origin, extent, and outcome of genetic variation, and provide researchers with a framework to understand these variations in order to further study of cancer. Single-cell gene expression data of non-small cell lung cancer showed that when FBXO17 (a negative modulator of glycogenase kinase 3β (44)) abundance was not under control, it regulated cell proliferation and survival by regulating Akt and ERK kinase activation, thus the potential role of F-box protein in regulating tumorigenesis was proposed (45). Another study performed identified knockdown of RAD51AP1 significantly suppressed tumor volume and prolonged survival in an intracranial EGFRvIII-positive glioma model by analyzing scRNA-seq data from wild-type EGFR cells and mutant EGFRvIII cells (32). ScRNA-seq data also revealed that the known nuclear hormone receptor retinoic-acid-receptor-related orphan receptor gamma (RORγ) in pancreatic cancer stem cells which drives T cell differentiation is up-regulated during the progression of tumor and its pharmacological inhibitory effect leads to significant defect in the growth and increase in survival rate of pancreatic cancer (14).

Genotypic analysis of gene expression variation by scRNA-seq is a useful supplement to the existing methods. Fan J developed a new HoneyBADGER method, which is used together with scRNA-seq to identify loss of heterogeneity and changes in copy number at the single-cell level. By examining data from patients with multiple myeloma (MM), the researchers found that although major gene subclones do show distinct transcriptional features conducive to tumor progression, other prominent aspects independent of the transcriptional heterogeneity of the gene sub-clonal structure may be driven by other mechanisms, including potential variations in epigenetic status or microenvironment (46). Meanwhile, Fasterius E. has also developed a single-cell variant analysis method. This method can be used to compare and cluster cells based on the genetic variation in single nucleotide variants. It also highlights the genetic heterogeneity of the tumor core, heterogeneity between metastases and high levels of variation in driver genes (47). This approach represents a considerable extension of scRNA-seq functionality, allowing researchers to take advantage of all the data generated from their experiments.

### Explore the Trajectory of Cell and Gene Development

The effects of molecular randomness, microenvironment and cell behavior usually lead to significant heterogeneity of cell population, thus blurring the dynamic biological principles that regulate cell state transitions. Single-cell high-throughput technology provides a way to discover these states and their transitions (48). There are many algorithms used in scRNA-seq that can infer the trajectory of a cell. A newly released algorithm, CellRouter (48), is very powerful in modeling the trajectory between the early cell state and the transitional cell state during cell differentiation. As another method of trajectory inference, STREAM can accurately reconstruct complex development trajectories (49). In the absence of surface markers, researchers have also developed genotyping of transcriptome (GoT), which has been used to study how somatic mutations disrupt the complex process of hematopoiesis in humans (50).

### Exploring the Developmental Trajectory of Cancer Cells

Nguyen QH and his colleagues used scRNA-seq and Monocle to analyze the transcriptome of mammary epithelial cells in seven patients undergoing mammoplasty, generated continuous pedigree levels for the Pseudotemporal reconstruction of differentiation trajectory, and linked a basic pedigree closely to two branches of the differentiated lumen pedigree (20). It provided evidence for determining the steady-state differentiation trajectory of adult breast and the origin of different subsets of basic and lumen pedigree. ScRNA-seq identified and characterized tumor plasma cells in low burden disease environment, such as asymptomatic precursor monoclonal gamma globulin disease (MGUS), with high sensitivity and confidence throughout the clinical progression from normal plasma cells in multiple myeloma. And a study suggested using direct molecular detection to track the pathogenesis of early multiple myeloma (51). ScRNA-seq results will provide resources for identifying changes during cancer progression, a basis for promoting cancer prevention strategies, and methods for early cancer detection.

### Exploring the Mutation Trajectory of Oncogenes

Despite detailed information on cancer variants and the frequency of clones, the order of acquisition of these mutations is unknown. The order of mutations in the cells can be elucidated by scRNA-seq. For example, CD34 + CD38 − pluripotent progenitor cells and bone marrow cells were analysed by scRNA-seq. It has been revealed that the loss of the fusion gene CDKN2A/B appears in the late stages of leukemia, while the mutation of NOTCH1 is a relatively late event (34). Depending on the order in which mutations are acquired, patients can have different clinical manifestations and response to treatment. In addition, scRNA-seq analysis has observed FOXP3 + malignant T cells and GATA3 + or IKZF2 +(HELIOS)tumor cells that are transformed from FOXP3 + T cells during cloning and evolution of Sézary, an aggressive form of cutaneous T-cell lymphoma (52). Similarly, a group of gastric precancerous lesions and early gastric cancer (EGC) specific marker genes were identified by scRNA-seq, for example, OR51E1 is a unique endocrine cell marker in the early stages of malignant lesions, and HES6 may mark the goblet cell cluster, which may be helpful for early identification of metaplasia (22). These genes have clinical value for predicting the early stages of cancer.

## Progress in the Application of scRNA-seq in tumor Microenvironment

**Figure 2:**
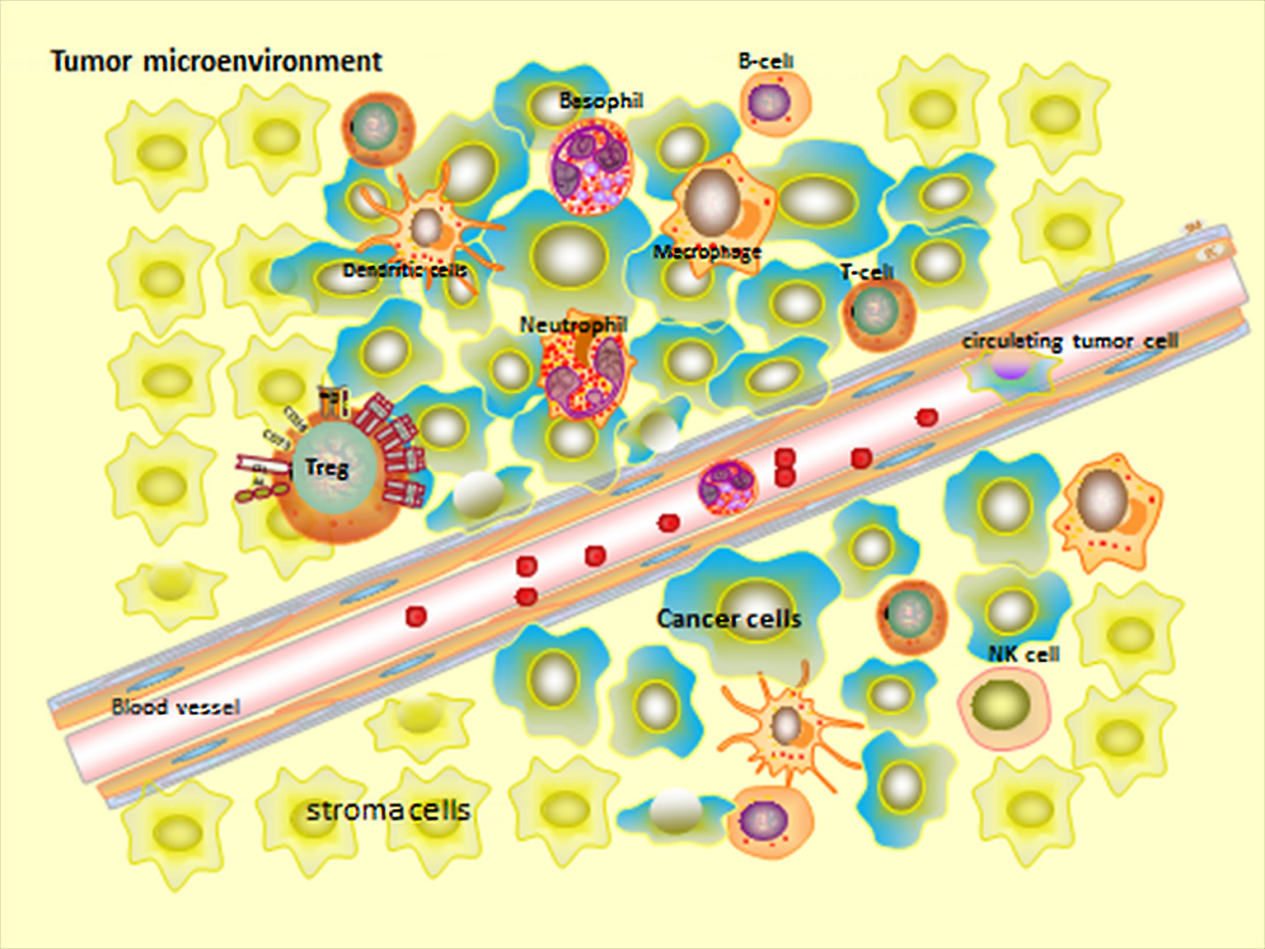
Tumor microenvironment.

Epithelial tissue tumors comprise complex and heterogeneous cell types from different sources, which can be divided into two categories: cancer cells originated from epithelial tissue and stroma cells. Stroma cells can be divided into: Infiltrating immune cells (IIC), cancer-related fibroblasts (CAF), and angiogenic vascular cells (AVC) (**Figure 2**) (53). Besides these, Jordan A. Ramilowski demonstrated that the normal function of postnatal animals is tightly controlled by intercellular communication and widely depends on the interaction between secreted ligands and cell surface receptors in tumor microenvironment (54, 55).

The infiltrating immune cells of tumor are also highly heterogeneous (56). Although cell signal pathway analysis and computational deconvolution analysis may indicate the main infiltrating cells, the results are not detailed enough (57). Methods to study the heterogeneity of tumor stroma cells also include laser dissection/capture and flow cytometry. These methods require prior knowledge of the specific marker genes to be visualized, but the blocking effects of marker genes on endothelial cells (EC), such as DLL4 and VEGF, make it difficult to be labeled (58). However, scRNA-seq results showed the role of VEGF and DLL4-Notch signal transduction can be detected in determining EC phenotype of tumors (46).

Microenvironmental cell types and gene changes are described at unprecedented resolutions by scRNA-seq for a variety of cancers at the single cell level, such as melanoma (59), glioma (13, 21), breast cancer (17), head and neck cancer (36), pancreatic cancer (31), and lung cancer (33). Some scientists have used scRNA-seq to construct an immune map of cancer in TME, for example, Elham Azizi constructed an immune map of breast cancer by analyzing immune cells from eight types of breast cancer and normal tissues, blood and lymph nodes (60), and Zheng isolated T cells from tumors, adjacent normal tissues, and peripheral blood to depict the immune map of hepatocellular carcinoma (30). Study also found that Prkar1a mutations in tumors result in dramatic changes in the genetic program of cancer cells through scRNA-seq, thus reshaping the microenvironment of the tumor (61).

### Tumor-Infiltrating T-lymphocytes

CD4+ regulatory T cells (Tregs) expressing transcription factor FOXP3 are highly immunosuppressive, and in malignant tumors, they promote cancer progression by suppressing antitumor immunity (62). Manu P. Kumar identified and compared the ligand-receptor interactions in six homologous mouse tumor models (B16-F10 melanoma, EMT6 breast cancer, LL2 lewis lung cancer, CT26 colon cancer, MC-38 colon cancer and Sa1N fibrosarcomas model) using scRNA-seq data and further quantified ligand-receptor interactions between T cell subsets and their relationship with immune infiltration (15). ScRNA-seq showed that Tregs and CD8 T cells were more abundant in primary liver tumors than in other tissues, but the majority of these T cells were in a depleted state, thus revealing why tumor cells escape immune surveillance (30). A class of FOXP3 suppressor T cells has been found in exhausted CD8 + T cell subsets, suggesting that depleted T cells may further develop into FOXP3 T cells (30).

ScRNA-seq can find many genes associated with TIL-T. De Simone M et al. found that several immune checkpoints and their ligand transcripts in tumor-infiltrating Treg cells were up-regulated in colorectal cancer or non-small cell lung cancer, and the specific signaling molecules were expressed on cell surface, such as interleukin-1 receptor 2 (IL1R2), programmed death (PD)-1Ligan1, PD-1ligand2, and CCR8 chemokines (63). TIL-T from follicular lymphoma (FL) also co-express genes with immune checkpoint molecules, such as CEBPA and B2M genes (16). Notably, the characteristic genes of Treg cells are highly expressed in whole tumor samples, for example, LAYN, MAGEH1 and CCR8 are associated with poor prognosis (63). Γδ T lymphocytes account for about 1% of human peripheral blood monocytes and have important anticancer functions. ScRNA-seq data can detect human γδ T lymphocytes from a large complex mixture of cells and specifically detect their T cell receptor (TCR) subsets, including TCRvδ1 and TCRvδ2. In human cancers, γδ TILs mainly express TCRvδ1, which is much less in tumor than in blood and has no correlation with the abundance of αβTIL (64). These findings provide a basis for an in-depth understanding of the molecular nature and function of human tumor-infiltrating Treg cells.

### B Lymphocytes

The number of B lymphocytes in tumor tissue is large, but the type of B cells in tumor tissue and the presence or absence of subtypes are unknown. A scRNA-seq study of lung cancer showed nine clusters of B cells. Six of these are enriched in tumors: follicular B cells that express high levels of CD20 (MS4A1), CXCR4 and HLA-DR (class 1 and 2), plasma B cells that express immunoglobulin (class 3 and 6), and mucosa-associated lymphoid tissue-derived (MALT) B cells that express immunoglobulin A, M and JCHAIN (class 5 and 7), demonstrating B cell heterogeneity in tumor tissue (65). In another study, the researchers explored a potential transcriptional network of follicular B-cell lymphoma through single-cell transcriptome. Based on gene expression, normal immune subsets and malignant B cells in each lymphoma were distinguished. Malignant B cells exhibit suppression of immunoglobulin (Ig) light chain (Igκ or Igλ) expression, upregulation of BCL2, FCER2, CD52 genes, and downregulation of major histocompatibility II genes. Interestingly, a mosaic of malignant B cell subclone coexisting in FL was found (16). ScRNA-seq can infer the B-cell population that other analytical methods cannot detect.

### Cancer-Related Fibroblasts

In some studies, the heterogeneity of cancer-related fibroblasts (CAFs) of various types of cancers has been demonstrated by scRNA-seq, and different subsets affect tumor microenvironment. For instance, Baryawno N demonstrated that one of the five fibroblast subpopulations in bone marrow expressed Cxcl12, and the fibroblasts expressing Cxcl12 were associated with invasive solid tumors (10). In colorectal cancer analyzed by scRNA-seq, there are two different CAF subtypes, CAF-A and CAF-B. Experimental results show that only CAF-A cells express fibroblast activation protein (FAP), a membrane-serine protease expressed only in CAF, so targeted treatment of FAP can be carried out based on the heterogeneity of CAF (65). Seurat divided CAFs from COLO205 tumor into six clusters and three different subpopulations, which were fibroblast-like cells, smooth muscle-like cells, and peripheral cells, respectively. It was found that two genes, Notch3 and Angpt2, which may be involved in angiogenesis, were highly expressed in peripheral cells. CAF can promote tumor angiogenesis by secreting feedback molecules during local hypoxia, and may be resistaant to angiogenic drugs (58).

In addition to immune cells themselves, CAF also plays a crucial role in immune escape and cancer metastasis. Studies have shown that upregulation of epithelial-mesenchymal transition (EMT)-related genes is driven by changes in expression in fibroblasts in colorectal tumors (65). In head and neck cancer, tumors with high CAF scores and high p-EMT scores have a particularly high tendency to metastasize, which may reflect that paracrine signaling between CAF and malignant cells can promote lymph node metastasis (36). In addition, it was found that a group of genes specifically expressed in CAF and closely associated with T cell infiltration included a variety of complementary factors (c1s, c1r, c3a, cfb and c1nh) in a study of melanoma. These results suggest that complement activity may be related to the regulation and recruitment of T cell-mediated anti-tumor immune responses, which provides clues for further study of the cellular and molecular mechanisms by which CAF recruits T cells (59).

### Tumor Infiltrating Myeloid Cells

TIMs contain monocytes, macrophages, dendritic cells, and neutrophils, which can diversify into a variety of states that can promote or limit tumor growth and become key regulators. ScRNA-seq was used to localize TIMs in patients with non-small cell lung cancer (NSCLC) and to analyze TIM in mice. Twenty-five TIM states were found, most of which could be found in different patient samples (66). Moreover, changes in myeloid cell population in tumors originated from the functional branch point of the infiltrating circulation affect monocytes, rather than the reprogramming of macrophages in mature tumor (67).

Zhu YP and colleagues used a scRNA-seq approach to show that mononuclear neutrophil progenitor cells (hNeP) significantly increase tumor growth after metastasis. In addition, hNeP is found in the blood of patients who have recently been diagnosed with melanoma, suggesting that hNeP can be used as a biomarker for early cancer detection, especially for cancers in which neutrophil levels and function are important (68). Tumor-associated macrophages (TAM) originate from myeloid precursors (69). TAMs, commonly known as polarized M2 macrophage populations, are cells with immunosuppressive and tumorigenic functions (70) and express arginase 1 (Arg1). It was found that there are two morphological subsets, Arg1+ TAM and Arg1- TAM, and that the pharmacological inhibitory effect of Arg1 is not synergistic with anti-programmed cell death 1 (aPD-1) therapy (71). Phenotypic differences in TAM of different lineages are distinguished by the scRNA-seq of human glioma. Hematogenous TAM is abundant in peripheral blood vessels and necrotic areas before treatment and significantly infiltrates gliomas, and its gene markers are related to the survival rate of gliomas and its infiltration varies with the subtypes of glioma. TAM preferentially expresses immunosuppressive cytokines (21). Moreover, autocrine interactions between glioma cells were identified. In addition, IL-8 is mainly expressed by TAM, and its receptor SDC1 is highly expressed in glioma stem cell-like cells (55).The results suggest that immunotherapy with immunosuppressive blood-derived TAM should be selected as a target.

## Research on Circulating Tumor Cell

Circulating tumor cell (CTC) not only provides an important mechanism for cancer metastasis, but also offers the possibility of diagnosing and monitoring cancer simply (72). In addition, repeated and quantitative analysis of nucleic acids in CTC can help to understand changes in clonal composition over time, enabling dynamic treatment (73). Studies have demonstrated the advantages of single-cell transcriptional profiling in detecting CTCs provide a new tool for the development of cancer biomarkers using liquid biopsy technology.

Because it is not feasible to evaluate IGF2 disorders (a kind of carcinogenic driver) by detecting tumor mutations alone, scRNA-seq of CTC is an excellent tool for detecting non-mutable drug-related gene abnormalities, such as IGF2 overexpression (28). CTC gene expression profiles of patients with Alveolar Rhabdomyosarcoma (ARMS) were analyzed by scRNA-seq. Of the top 150 genes with the greatest difference, 70 genes were expressed at a significant level in the CTC population. These genes have previously been shown to be associated with metastasis, but have never been detected in ARMS (35). This may suggest novel genes for ARMS diagnosis.

ScRNA-seq of breast cancer CTCs identified them into two types: one with estrogen responsiveness and another with EMT characteristic (74). Another study used a scalable hydrodynamic scRNA-seq bar coding technique called Hydro-Seq to resolve the contamination of blood cells. They identified cells that express markers of epithelial/mesenchymal cell state transitions and detected drug targets of breast cancer CTCs (75). In the future, scRNA-seq is expected to help physicians select appropriate anti-cancer drugs and treatments, and monitor the progression of the disease and the therapeutic effect at any time, which will help personalized treatment for patients.

## Research in Cancer Therapy

Chemotherapy, targeted therapy and immunotherapy have always been vital weapons in the fight against cancer. Due to the importance of ligand-receptor interactions for patient prognosis, such as ipilimumab, an immunosuppressive agent targeting CD28 or CTLA4, and pembrolizumab and nivolumab targeting PD1 or PDL1, therapeutic agents targeting immunization checkpoints have become a promising approach in clinical treatment (76). High-dimensional analysis can not only help to identify the best combination of a variety of available immunotherapy drugs, but also identify potential new reactive biomarkers, so that cancer immunotherapy is more effective, specific, and safe than the previous one available to us.

Cancer cells will develop resistance and cross-resistance to a variety of chemotherapeutic drugs that have unrelated functions and structures after exposure to chemotherapeutic drugs (77). In fully active and symptomatic diseases, the frequent identification of the precise molecular characteristics and polyclonal structure of malignant state by scRNA-seq provides a better understanding of the mechanisms of resistance after treatment (51). ScRNA-seq studies have also revealed a high-resolution picture of drug-resistant cells to immune checkpoint inhibitors (ICIs), which provides a framework for studying cell-cell interactions and drug interactions in other tumor ecosystems.

### Targeted Therapy

Resistance to targeted BRAF inhibitors is widely existed in melanoma (78). Using scRNA-seq analysis and cluster assessment (SAKE) to track melanoma cells that have developed resistance to BRAF inhibitors, several BRAF inhibitor resistance markers have been obtained, and new resistance markers have been identified in very few cell populations before using drug (24). Some scRNA-seq data confirm that neural c stem cells (NCSC) are the major driving force for drug resistance. Application of ScRNA-seq to patient-derived melanoma minimal residual disease (MRD) of BRAF mutant xenotransplantation identified up to four different drug-resistant transcriptional states. One of them showed the NCSC transcriptional program driven mainly by the nuclear receptor RXRG. RXR antagonists reduced the accumulation of NCSC in MRD and delayed the development of resistance (23).

ScRNA-seq reveals the rationale for improving biomarkers and providing new treatments to patients, and suggests new therapeutic strategies to overcome drug resistance in immunotherapy.Two patients with metastatic Merkel cell carcinoma (MCC) treated with T-cell immunotherapy and immune checkpoint inhibitors (ICIs) were also studied with scRNA-seq. It was observed that the injected CD8 T cells infiltrated into the reduced MCC, and the tumor regression was mediated by supporting T cells. By targeting the selective transcription loss of human leukocyte antigen (HLA) under T-cell pressure to limit targeting the epitopes of Merkel cell polyomavirus (MCPyV), the difference from the genetic loss of HLA is that drug treatment may reverse the inhibitory action of tumor-specific HLA (25). Transcriptional inhibition of class I loci may be the basis of resistance to other immunotherapy (including checkpoint inhibitors). In addition, another study applied scRNA-seq and identified a malignant cellular program related to T cell rejection and ICI resistance prediction in melanoma. It demonstrated that CDK4/6 inhibitors can inhibit this program and make melanoma tumor in a mouse model sensitive to ICI (79).

### Chemotherapy

Therapies that target the epidermal growth factor receptor (EGFR) have variable and unpredictable effects in breast cancer. Cell subsets with EGFR inhibitory response to gefitinib were identified by scRNA-seq and EGFR^hi^ subsets showed enhanced stem cell-like characteristics in three negative breast cancer (TNBC) (18). Heterogeneous expression of EGFR is associated with the sensitivity to gefitinib, which provides a basis for further treatment planning.

In a TNBC mouse model, scRNA-seq data revealed that the hedgehog (Hh) signal from the fibroblast (CAF) binds to Hh ligands from tumor cells, which promotes FGF5 expression and collagen remodeling in the matrix, resulting in the production of cancer stem cells (CSCs) with chemoresistant phenotype. Matrix therapy with smoothened inhibitors (SMOi) for patient-derived xenografts can down-regulate the expression of CSC markers and make the tumor sensitive to Docetaxel, thereby significantly increasing the survival rate and reducing the rate of metastasis (19). At the same time, the hedgehog signal sent by CAF will be a new plastic mediator for CSC and a new therapeutic target for TNBC.

### Immunotherapy

In the present study, scRNA-seq addresses the challenges of insufficient understanding of the complexity of immune cell subtypes and possible differences in the immune system between species, which lays the foundation for studying the potential of immune cell as a target of immunotherapy in the future. ScRNA-seq shows that the effects of different immune checkpoint therapy (ICT) on monocytes/macrophages in tumors are particularly significant, and they are partially dependent on IFNγ and change over time, which leads to a high degree of plasticity and complexity of the cell population (67). These insights into the transcriptional, molecular, and functional changes that occur within the immune cells of major tumors after cancer immunotherapy strongly support the need to simultaneously consider both innate (e.g., macrophage) immunity and adaptive (e.g., CD4 and CD8 T cells) immunity to improve the efficacy of cancer immunotherapy.

ScRNA-seq can explore the effects of certain cytokines on immune cell development and discover some important novel immune cells that have not been revealed (80). For example, scRNA-seq and lineage tracing identified a population of TCF- 1+ Ly108+ PD-1+ CD8 T cells and revealed that TCF-1 mediated T-bet-to-Eomes transcription factor conversion in the culture of exhausted CD8 T cell (Tex) precursors, and PD-1 was identified as a protector of early TCF-1 subgroup (81). Congenital lymphoid cells (ILCs) are a newly identified family of innate immune cells. ScRNA-seq revealed that differentially expressed ikzf3 in human ILC1 is a known target of immunomodulatory drug (IMiD) -mediated degradation such as lenalidomide or pomalidomide, which increases the possibility that ILC may also be a IMiD mediated immunomodulatory target (82). Kubli SP et al. used single-cell transcriptome analysis to confirm that FCMR (a putative receptor for soluble IgM) that plays an important role in regulating immune responses during autoimmunity generally acts to limit DC maturation in the TME and then suppresses antitumor T cell responses (83). Blockage of FCMR and synergistic treatment with T cell-specific anti-PD1 in myeloid cells can inhibit the growth of B16 melanoma (83).

The blocking of the reactivation of immune response by PD-1, a marker of T-cell exhaustion, is becoming a promising cancer treatment. The recurrence of hematological malignancies after allogeneic stem cell transplantation (allo-SCT) limits the success of this approach. Because PD-1 expression may differ from that of non-transplant individuals, its blocking may lead to graft-versus-host disease (GVHD). The kinetics of T cell exhaustion and its relationship with leukemia recurrence were analyzed by scRNA-seq in patients undergoing allo-SCT. Although leukemia antigen-specific T cells do not overexpress PD-1, LAG3 and TIM3 are over expressed during relapse (26). So we could target LAG3 and TIM3 as a new therapy approach.

## Conclusion

Despite the many advantages of scRNA-seq, certain limitations and challenges of scRNA-seq cannot be avoided. In single-cell level studies, the main problem is that the starting amount of RNA is low and the reverse transcriptome is not easily amplified (46). Another major problem with scRNA-seq is the increase in impurity levels in the abundance of transcripts measured. At the same time, excessive transcript loss rates and random transcription events will result in a large amount of data, high variability, and complex expression distribution undetected. Therefore, it is important to distinguish low quality, high impurity samples with poor amplification or degradation in the process of library preparation (24). Data on the use of single-cell techniques to analyze the genome-wide transcriptome of CTCs are scarce. Due to the rarity of CTC in blood (about 0–10 CTCs in 7,000,000 nucleated cells), we need to enrich CTC in large numbers before single-cell sequencing. ScRNA-seq strongly implies the origin of tumors in isolated cells, but the low coverage and dispersion of these data make it impossible to clearly assess malignant characteristics, such as somatic mutations or chromosomal aberrations (28).

Single-cell-range technologies offer the advantage of measuring multiple molecules such as DNA, RNA, proteins, and chromatin, at high resolution. By isolating different types of molecules from a single cell simultaneously, they can be analyzed in parallel (11). For example, the combination of whole genome and whole transcriptome sequencing (scGT-seq) can help establish the relationship between genomic changes in cancer and their effects on immune cells (84), such as studies on gastric cancer and primary gastric tumors (85). A combination of single-cell DNA methylation and transcriptome sequencing (scMT-seq) found that Low-methylated regions (LMR) showed significant difference in methylation levels, which is consistent with their role as remote regulatory elements to control gene expression (84). ScTrio-Seq has further developed single-cell multi-omics technologies by combining three omics methods, genomics, transcriptomics, and epigenomics. The spatial information of individual cells in a tissue is usually lost during the isolation step, so individual cell sequencing data usually do not show how cells organize to achieve coordinated functions within the target tissue (26). The field of single-cell spatial transcription is under intensive investigation, and many new technologies have been developed to maintain or restore spatial information of sequenced single cells, such as seqFISH (sequential fluorescence *in situ* hybridization of RNA) (86), MERFISH (Multiplexed error-robust fluorescence *in situ* hybridization) (87), FISSEQ (fluorescent *in situ* sequencing) (88), or TIVA (Transcriptome *in vivo* analysis) (89). All of these methods can identify interactions between different cell types by examining genes expressed *in vivo* in the context of specific tissue structures.

ScRNA-seq technology can produce a lot of data, some current bioinformatics tools need to be used to analyze these data (90). Principal component analysis (PCA) was performed to reduce the dimensionality on the log transformed gene-barcode matrices of top variable genes. Cells were clustered based on a graph-based clustering approach and were visualized in 2-dimension using t-SNE. R package SingleR, a novel computational method for unbiased cell type recognition of scRNA-seq, is used to infer the cell of origin of each of the single cells independently and identify cell types (91). Using Monocle algorithm can measure a cell’s biological progression, which is called “pseudotime” (92). Further technical improvements in bioinformatics tools will greatly facilitate the applications of scRNA-seq.

The continuous development of single-cell transcriptome technology and its combination with multi-domain technologies and algorithms will bring new revolutions to next generation genome sequencing. At the same time, its unique role in the field of cancer will help us explain biological mechanisms that could not be elucidated before and greatly promote the development of precision medicine, bringing new breakthroughs in clinical diagnosis, treatment, and prognosis of patients.

## Author Contributions

JL wrote the manuscript and collected the literature. TX collected the literature and modified the manuscript. YJ collected the literature. BH proposed the amendments. YZ designed the content of the article and proposed amendments. All authors contributed to the article and approved the submitted version.

## Funding

The work was supported by the National Natural Science Foundation of China (81772772) and the Department of Science and Technology, Jilin Province, China (CN)(20190905004SF, 20170622008JC).

## Conflict of Interest

The authors declare that the research was conducted in the absence of any commercial or financial relationships that could be construed as a potential conflict of interest.
